# Supplementary material for: Chidamide plus Tyrosine Kinase Inhibitor Remodel the Tumor Immune Microenvironment and Reduce Tumor Progression When Combined with Immune Checkpoint Inhibitor in Naïve and Anti-PD-1 Resistant CT26-Bearing Mice
Source: Int J Mol Sci. 2022 Sep 14;23(18):10677. doi: 10.3390/ijms231810677 (PMC9504159; doi:10.3390/ijms231810677)
Supplement: Supplementary file 1 [file ijms-23-10677-s001.zip › ijms-1887742-supplementary.pdf]

Supplementary Material

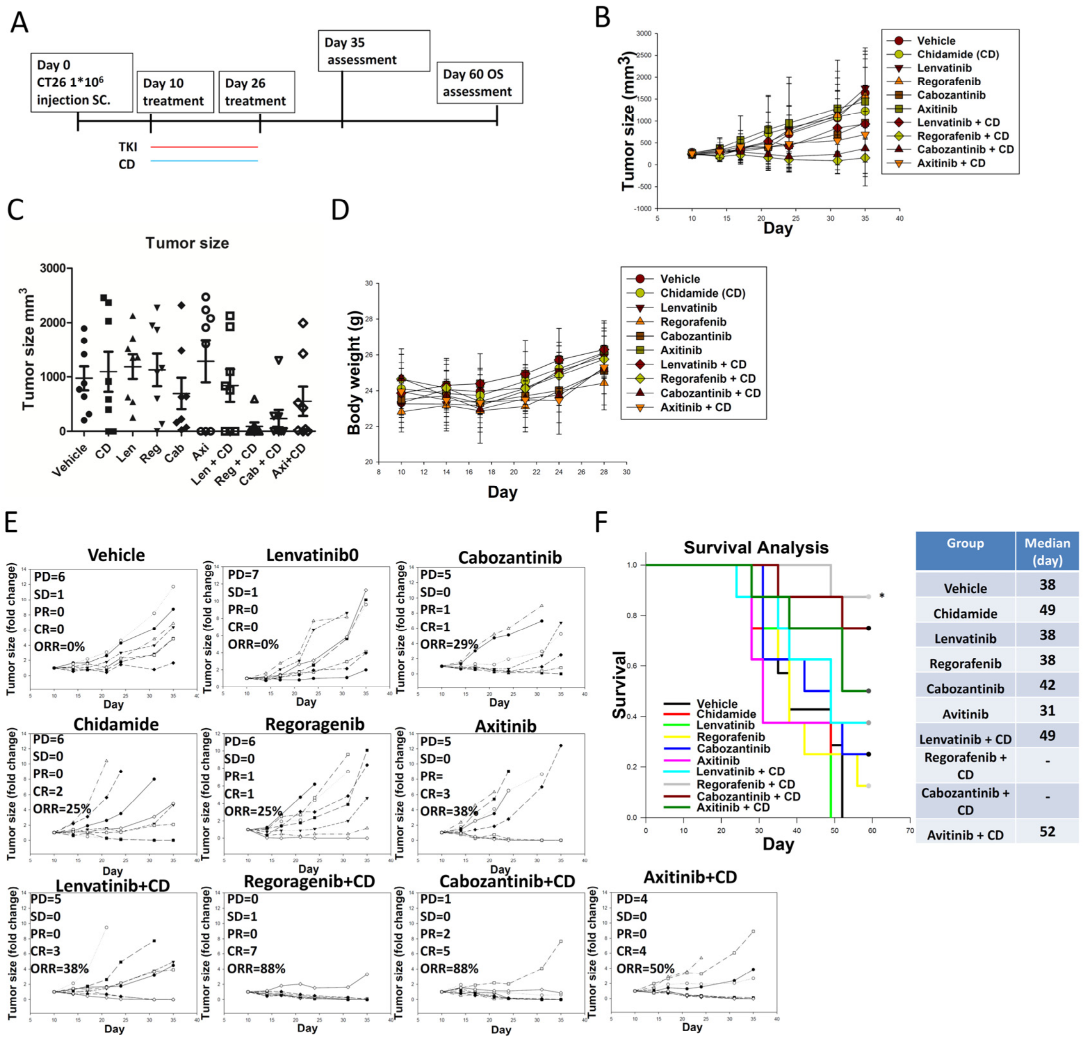

Supplementary Figure S1. (A) to (F) showed the results of efficacy comparison of Vehicle, Lenvatinib, Cabozantinib, Chidamide, Regorafenib, Axitinib, and 4 compounds combined with Chidamide-k30 in CT26 tumor-bearing mice. Balb/c mice bearing a CT26 tumor were treated with various therapeutic modalities as indicated. (A) Consecutive treatment schedule and total tumor volumes. (B) Tumor volume change. (C) Endpoint evaluated tumor volumes at D28. (D) Mice body weights. (E) Individual tumor volumes. (F) Animal survival rates. CT26 tumor-bearing mice were treated as indicated and euthanized at a tumor volume of 3000 mm^3 after tumor implantation. Data are given as mean ± SD; *P < 0.05, **P < 0.01, ***P < 0.001, one-way ANOVA with Tukey's test.*, compared to IgG; #, compared to PD-1. (n=7-8)

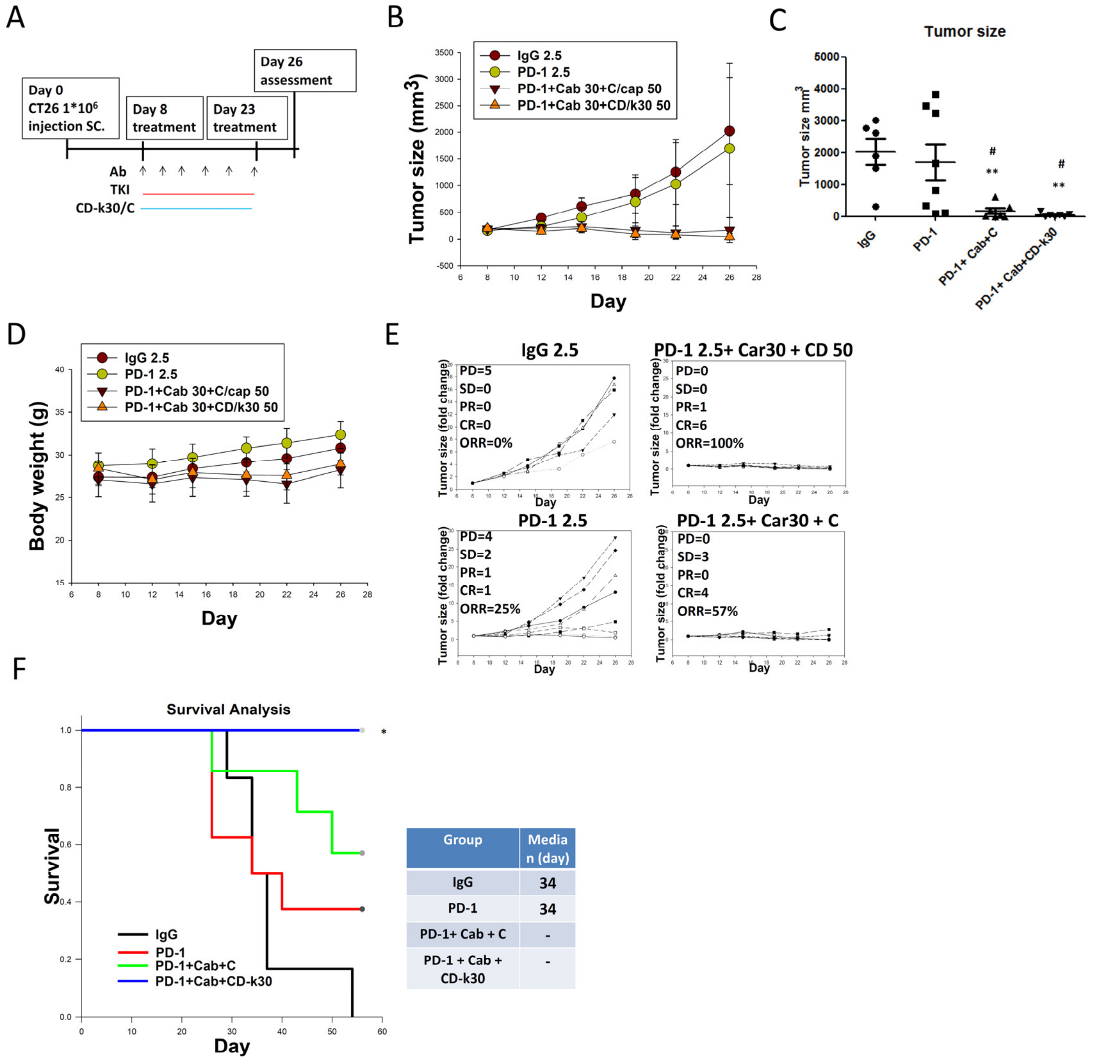

Group	Median (day)
IgG	34
PD-1	34
PD-1+ Cab + C	-
PD-1 + Cab + CD-k30	-

Supplementary Figure S2. (A) to (F) showed the results of therapeutic response of Cabozantinib plus Celecoxib or Chidamide-k30 combined with anti-PD-1 antibody in CT26 tumor-bearing mice. Balb/c mice bearing a CT26 tumor were treated with various therapeutic modalities as indicated. (A) Consecutive treatment schedule and total tumor volumes. (B) Tumor fold change. (C) Endpoint evaluated tumor volumes at D26. (D) Mice body weights. (E) Individual tumor volumes. (F) Animal survival rates. CT26 tumor-bearing mice were treated as indicated and euthanized at a tumor volume of 3000 mm^3 after tumor implantation. Data are given as mean ± SD; *P < 0.05, **P < 0.01, ***P < 0.001 , one-way ANOVA with Tukey's test.*, compared to IgG; #, compared to PD-1.

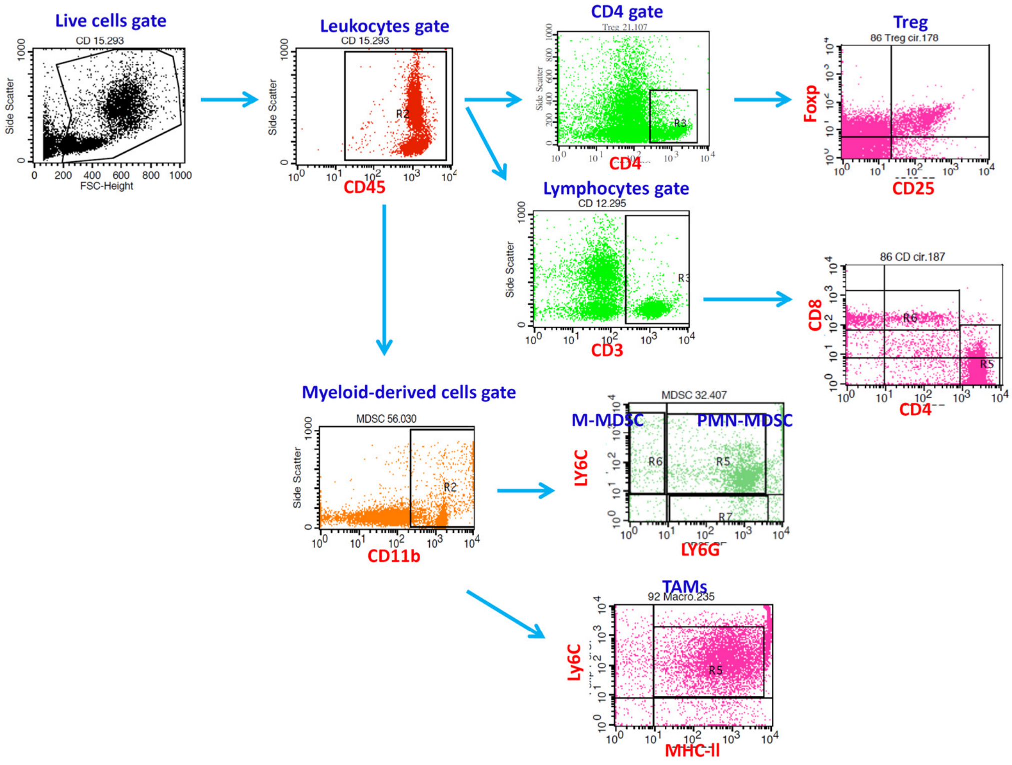

Supplementary Figure S3. Representative demonstrates FACS staining of Treg, CD4, CD8, MDSC, and TMA subsets in the peripheral blood cells and Tumor from CT26-bear mice.

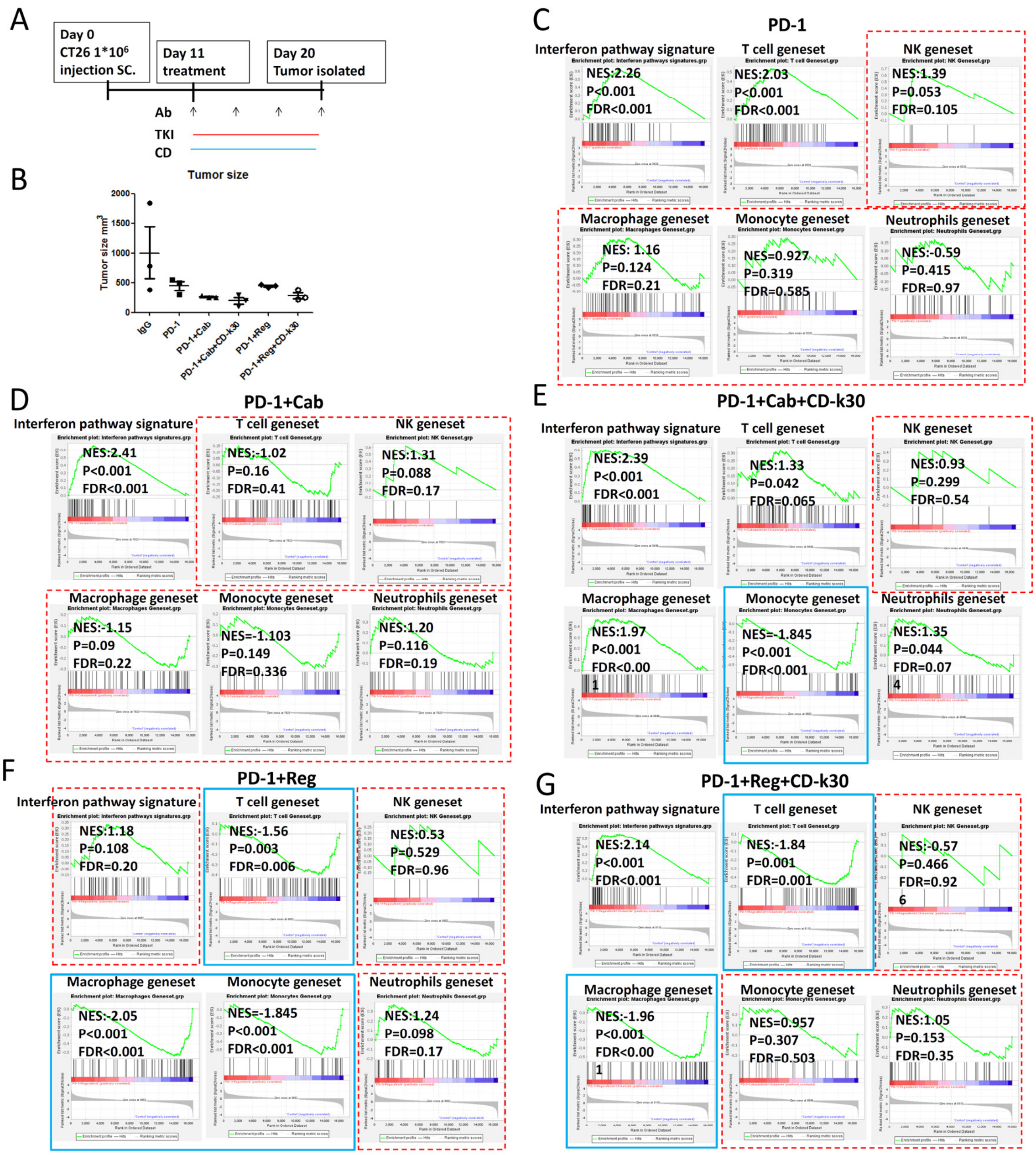

Supplementary Figure S4. Identification of target genes of anti-PD-1 Ab combined with Regorafenib/Cabozantinib plus Chidamide-k30 treatment,which significantly regulates gene expression in TME of CT26 tumors-bearing mice. Tumors were analyzed on day 20 after 9-day treatment for gene expression by RNA-seq. (A) consecutive treatment schedule and (B) Tumor sizes of each treatment group. (C) to (G) show Gene Set Enrichment Analysis (GSEA) in the regimens of anti-PD-1 Ab combined with Regorafenib/Cabozantinib plus Chidamide-k30, that significantly regulates gene expression in TME of CT26 tumors-bearing mice. The CT26 tumor-bearing mice were treated with various therapeutic modalities as indicated. Tumors were analyzed on day 20 after 9-day treatment for gene expression by

RNA-seq. GSEA of gene expression related to Interferon pathway signature, Macrophage geneset, Monocyte geneset, Neutrophils geneset, NK geneset, T cell geneset. (C) PD-1 Ab. (D) PD-1 Ab+Cab. (E) PD-1+Cab+CD-k30. (F) PD-1Ab+Reg. (G) PD-1+Reg+CD-k30. NES: normalized enrichment score; FDR: false discovery rates. Signature scores were calculated by mean log2 (TPM) of their respective member genes; P-values: Mann-Whitney test, two-tailed. TPM, transcripts per million; DGE, differential gene expression. When $P \geqq 0.05$, the GSEA analysis panel(s) will be outlined with red dotted line. When gene expression is down-regulated, the GSEA analysis panel(s) will be outlined with blue solid line.

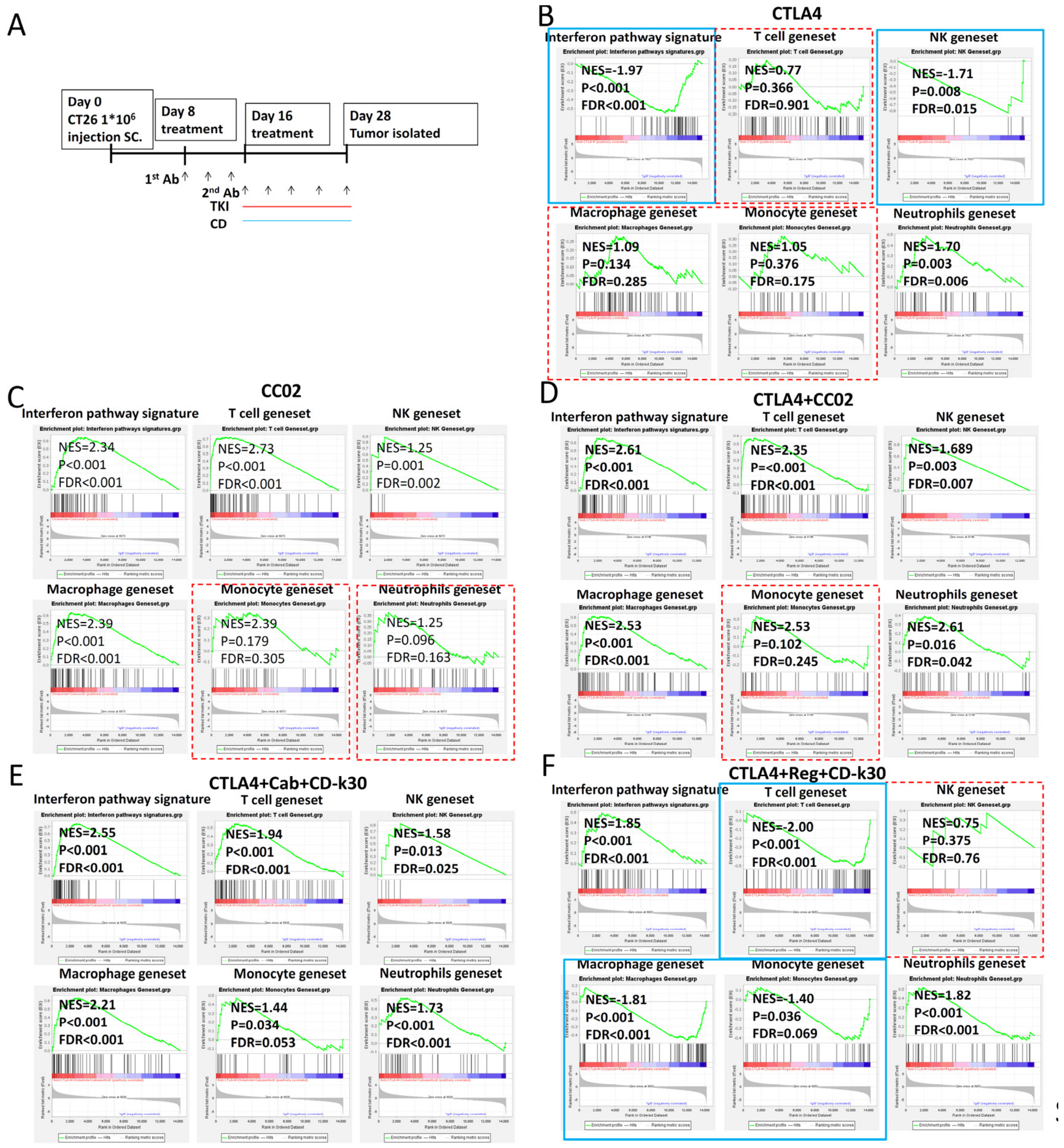

Supplementary Figure S5. Identification of target genes of second-line treatment with anti-CTLA-4 Ab combined with CC-02 or Regorafenib/Cabozantinib plus Chidamide-k30 that significantly regulates gene expression in TME of CT26 tumors-bearing mice. Tumors were analyzed on day 12 after starting second-line treatment for gene expression by RNA-seq. (A) consecutive treatment schedule (B) to (F) show Gene Set Enrichment Analysis (GSEA) in the regimens of anti-CTLA-4 Ab combined with Regorafenib/Cabozantinib plus Chidamide-k30 that significantly regulates gene expression in TME of CT26 tumors-bearing mice. Tumors were analyzed on day 28 after 12-day treatment for gene expression by RNA-seq. GSEA of gene expression related to Interferon pathway signature, Macrophage geneset, Monocyte geneset, Neutrophils geneset, NK geneset, T cell geneset. (B) anti-CTLA-4 Ab. (C) anti-CTLA-4 Ab+Cab. (D) anti-CTLA-4 Ab +Cab+CD-k30. (E)

anti-CTLA-4 Ab +Reg. (F) anti-CTLA-4 Ab +Reg+CD-k30. NES: normalized enrichment score; FDR: false discovery rates. Signature scores were calculated by mean log2 (TPM) of their respective member genes; P-values: Mann-Whitney test, two-tailed. TPM, transcripts per million; DGE, differential gene expression. When P $\geqq$0.05, the GSEA analysis panel(s) will be outlined with red dotted line. When gene expression is down-regulated, the GSEA analysis panel(s) will be outlined with blue solid line.

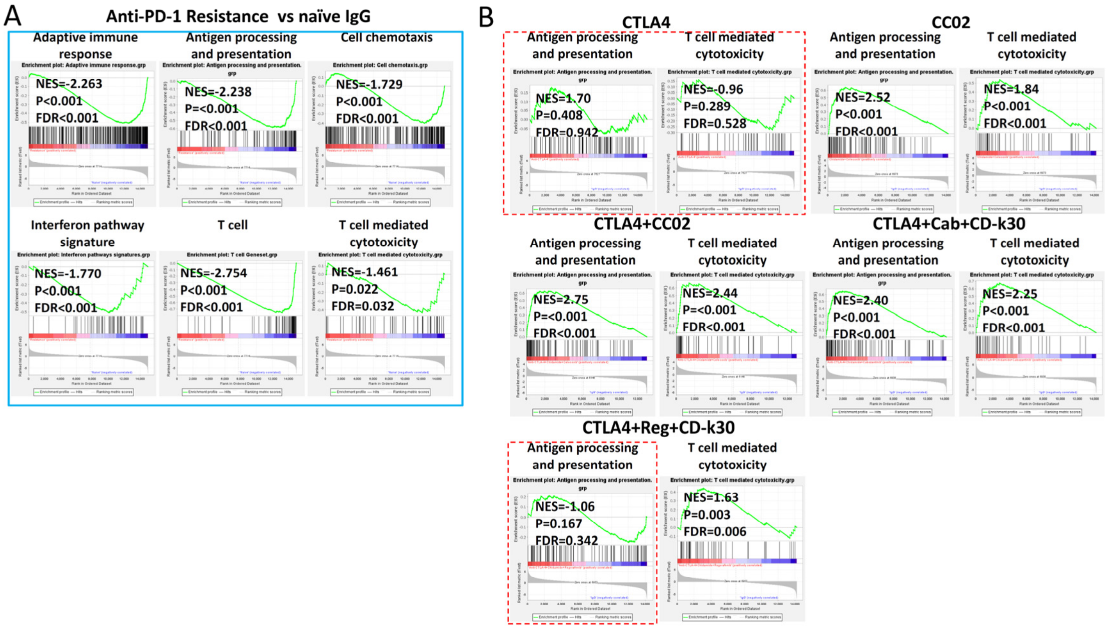

Supplementary Figure S6. (A) to (B) show Gene Set Enrichment Analysis (GSEA) for non-responsive first-line anti-PD-1 Ab and different second-line treatment regimens that significantly regulates gene expression in TME of CT26 tumors-bearing mice. Tumors were analyzed on day 28 after 12-day treatment for gene expression by RNA-seq. (A) GSEA of gene expression related to Adaptive immune response, Antigen processing and presentation, Cell chemotaxis, Interferon pathway signature, T cell, T cell mediated cytotoxicity gene set in non-responsive to first-line anti-PD-1 Ab vs naïve IgG treatment. Non-responsive to first-line anti-PD-1 Ab treatment is shown in Figure 6B. (B) GSEA of gene expression related to Antigen processing and presentation and T cell mediated cytotoxicity gene set in anti-CTLA-4 Ab, CC02, anti-CTLA-4 Ab +CC02, anti-CTLA-4 Ab +Cab+CD-k30, and anti-CTLA-4 Ab +Reg+CD-k30 vs naïve IgG treatment. NES: normalized enrichment score; FDR: false discovery rates. Signature scores were calculated by mean log2 (TPM) of their respective member genes; P-values: Mann-Whitney test, two-tailed. TPM, transcripts per million; DGE, differential gene expression. When P≧0.05, the GSEA analysis panel(s) will be outlined with red dotted line. When gene expression is down-regulated, the GSEA analysis panel(s) will be outlined with blue solid line.
